# Most patient conditions do not a priori debilitate the sensitivity of thoracic ultrasound in thoracic surgery-a prospective comparative study

**DOI:** 10.1186/s13019-021-01454-6

**Published:** 2021-04-13

**Authors:** Thomas Galetin, Julika Merres, Mark Schieren, Benjamin Marks, Yves Haffke, Jerome Defosse, Frank Wappler, Aris Koryllos, Erich Stoelben

**Affiliations:** 1 Lungclinic, Thoracic Surgery, University Witten/Herdecke, Medical Centre Cologne-Merheim, Ostmerheimer Str. 200, 51109 Cologne, Germany; 2 Department of Anaesthesiology and Intensive Care Medicine, University Witten/Herdecke, Medical Centre Cologne-Merheim, Cologne, Germany; 3 Department of Radiology, University Witten/Herdecke, Medical Centre Cologne-Merheim, Cologne, Germany

**Keywords:** Lung ultrasound, Pneumothorax, Thoracic surgery, Sensitivity

## Abstract

**Background:**

The few existing studies on the accuracy of lung ultrasound in the detection of a postoperative pneumothorax after thoracic surgery differ in the sonographic technique and the inclusion criteria. Several conditions are considered unfavourable in the sonographic examination of the lung. We aim to test these conditions for their impact on the diagnostic accuracy of lung ultrasound.

**Methods:**

We compared lung ultrasound and chest roentgenograms for the detection of a pneumothorax after lung-resecting surgery in two prospective trials (register ID DRKS00014557 and DRKS00020216). The ultrasound examiners and radiologists were blinded towards the corresponding findings. We performed posthoc subgroup analyses to determine the influence of various patient or surgery related conditions on the sensitivity and specificity of ultrasound in the detection of pneumothorax.

**Results:**

We performed 340 examinations in 208 patients. The covariates were age, gender, body mass index, smoking status, severity of chronic obstructive pulmonary disease, previous ipsilateral operation or irradiation, thoracotomy, postoperative skin emphysema, indwelling chest tube and X-ray in supine position. In univariate analysis, an indwelling chest-tube was associated with a higher sensitivity (58%, *p* = 0.04), and a postoperative subcutaneous emphysema with a lower specificity (73% vs. 88%, *p* = 0.02). None of the other subgroups differed in sensitivity or specificity from the total population .

**Conclusions:**

Most of the patient- or surgery related conditions usually considered unfavourable for lung ultrasound did not impair the sensitivity or specificity of lung ultrasound. Further studies should not excluce patients with these conditions, but test the accuracy under routine conditions.

**Trial registration:**

DRKS, DRKS00014557, registered 06/09/2018, https://www.drks.de/drks_web/navigate.do?navigationId=trial.HTML&TRIAL_ID=DRKS00014557 and DRKS00020216, registered 03/12/2019, https://www.drks.de/drks_web/navigate.do?navigationId=trial.HTML&TRIAL_ID=DRKS00020216

**Supplementary Information:**

The online version contains supplementary material available at 10.1186/s13019-021-01454-6.

## Introduction

Thoracic surgery is inevitably associated with the frequent use of postoperative thoracic imaging, particularly chest roentgenograms. Very rarely do they have a therapeutic consequence [1, 2]. There are efforts to reduce the number of routine X-rays by identifying criteria which clearly indicate thoracic imaging [1, 3–12]. However, chest roentgenograms remain the standard practice, even in an uneventful postoperative course. Clinical examination alone may miss major abnormities [9]. While ultrasound has been proven to surmount chest X-ray in the detection of a pneumothorax in large-scaled meta-analyses from intensive care medicine and traumatology [13–15], there are only few and contradictory data from thoracic surgery. There are no standards of chest ultrasound examination in thoracic surgery, and the existing trials vary extensively in the ultrasound technique and inclusion criteria. Many trials, surgical and medical, omit relevant patient groups because they presumably are unsuitable for chest ultrasound, for example, patients with chronic pulmonary diseases, previous surgery or postoperative subcutaneous emphysema. However, these patients represent the majority of the daily routine patient population in a thoracic surgery department. Furthermore, there is not any evidence that these conditions should indeed be avoided. Therefore, we have recently conducted a prospective observational trial which compared a standardised lung ultrasound with p.a. upright chest X-ray after chest tube removal following thoracic surgery [16, 17]. We have refrained from excluding the “difficult” patients to evaluate a pragmatic, real-life accuracy of chest ultrasound. While overall sensitivity was low (48% on the first postoperative day and 32% after removing the chest tube), we still found a high usefulness of ultrasound to safely recognise all relevant pneumothoraces (≥ 3 cm, sensitivity 100%) and a high congruity of the hypothetical sonography-based treatment recommendation with the actual routine-based patient management (97%). We now report the results of posthoc subgroup analyses to illustrate the impact of those conditions usually considered inappropriate for lung ultrasound on imaging and test quality.

## Methods

### Study design

We analyse data from a prospective observational diagnostic accuracy study (SONOR, trial ID DRKS00014557), and its methodically identical successor SONOR2, trial ID DRKS00020216, which both aim to compare ultrasound (index test) with routine chest roentgenograms (reference test) to detect a pneumothorax (outcome) after thoracic surgery (target population). The studies were approved by the ethics committee of the University of Witten/Herdecke and registered at the WHO-conform German Clinical Trials Registry. Patients received a standardised ultrasound examination on the same day as routine chest roentgenograms which were performed on the first postoperative day and after removing the chest tube. There were no ultrasound-related exclusion criteria with regard to patient selection, but sonographic examination depended on the availability of the sonographer. There were no ultrasound-related exclusion criteria, so that all patients, even if deemed “inappropriate” for chest ultrasound, were included in the analysis. The ultrasound examiner and the dedicated radiologist were blinded towards the complementary results. The primary endpoint of the study was the sensitivity of lung ultrasound for a residual pneumothorax of any size after chest tube removal following non-cardiac thoracic surgery. However, for the purpose of this posthoc analysis, all examinations, on the first postoperative day as well as after chest-tube removal, were considered.

### Clinical investigation

The ultrasound examination followed the principles and definitions of the BLUE-protocol [18]. Convex and linear probes were used on the anterior chest wall of a (semi-)recumbent patient to detect a pneumothorax. Wound dressings, ECG-electrodes etc. were not removed, the probe position was adapted if necessary. A pneumothorax was ruled out if any of lung-sliding, B-lines, I-lines, consolidations or lung-pulse was detected. A pneumothorax was defined as a) visible lung-point (accuracy 100%) or b) absence of any rule-out signs (accuracy 96%) [19]. Inconclusive findings were considered negative for pneumothorax for the purpose of diagnostic accuracy analysis. Chest X-ray was performed in p.a.-direction in erect position whenever possible; in immobile patients, supine a.p. roentgenograms were performed.

### Patients

Consecutive patients undergoing lung-resecting surgery except for pneumonectomy were enrolled after giving informed consent. All adult mentally and legally competent, non-pregnant patients were included in the study. There were no ultrasound-specific exclusion criteria.

### Statistics

X-ray is an imperfect reference test *R* but with 100% specificity, thus, the sensitivity se of the index test *I* can be calculated by *se* = P(*I* +  │ *R*+)/(P(*I* +  │ *R*+) + P(*I* −  │ *R*+)).

The true specificity *sp* can be approximated, lying between the observed specificity *sp*_*observed*_ = P(*I*−| *R*−)/(P(*I*−| *R*−) + P(*I*+| *R*−)) and 100% [20].

To estimate the impact of potential explanatory variables which are supposed to impair the accuracy of ultrasound, we performed the subsequent analyses:

First, we identified factors which may influence the sensitivity of postoperative lung-ultrasound regarding a pneumothorax from literature, especially from (cardio-)thoracic surgery. These were: obesity [21, 22], subcutaneous emphysema [21–23], chronic obstructive pulmonary disease [24], severe lung emphysema [23], old patients [21], inserted chest tube [25], pleurodesis [25, 26] (for instance, by previous surgery), immobile or weak patients because they cannot be examined in sitting position [23]. The impact of most of these covariates could be reflected by our data, except for the latter, because all our patients were examined by ultrasound in (semi-)recumbent position.

Second, we computed the sensitivity and observed specificity with and without those “difficult” conditions.

Third, we made a univariate analysis for these covariates, comparing the subgroup with false-negative ultrasound with the remaining population; we computed contingency tables and performed student’s t-test for continuous independent and Chi-squared test for categorical data.

Binomial data are reported as proportions, multicategorical data as mode, continuous data as mean and standard deviation.

### Data management and analysis

Study data were captured with Castor EDC [27], a web-based system fully compliant to the rules of good clinical practice and privacy protection. Statistical analysis was performed with R, version 3.6.1 [28] and the library “mada” [29].

## Results

We performed 340 examinations in 208 patients; 132 patients were examined twice, on the first day after surgery with an indwelling chest tube and after removing the chest tube. Fifty-five percent were male, the mean age was 64.6 ± 11.3 years, 280 patients (82%) were current or former smokers with overall 40.9 ± 24.1 packyears. Thirty-seven patients had a COPD GOLD 1, 108 GOLD 2, 23 GOLD 3, 5 GOLD 4. Sixty-eight patients (20%) had an ipsilateral pretreatment, i.e. tumour irradiation or previous lung surgery. There were 226 anatomic, 94 wedge, 13 extended resections (with chest wall or diaphragm) and 9 other operations with pulmonary resection.

We analysed 11 potential factors which could impair chest ultrasound: gender, higher age, elevated BMI, advanced COPD, smoking, previous ipsilateral surgery or irradiation, thoracotomy, indwelling chest tube, postoperative soft tissue emphysema, X-ray in supine position.

**Fig. 1:**
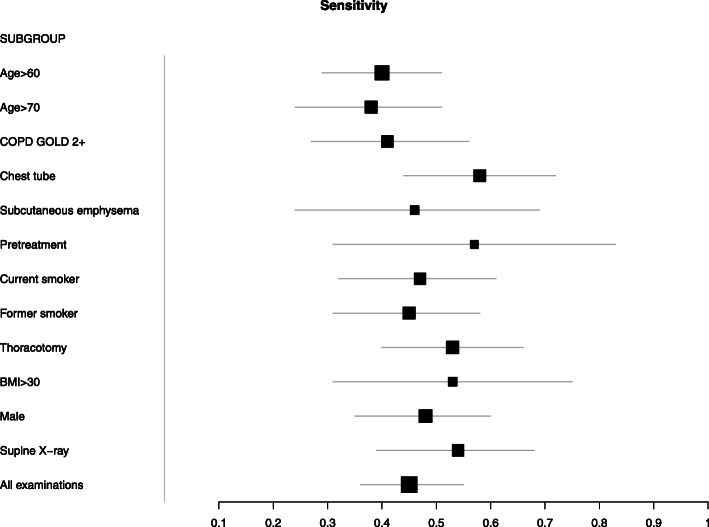
Forest plot of sensitivity of lung ultrasound in dependence of different covariates

**Fig. 2:**
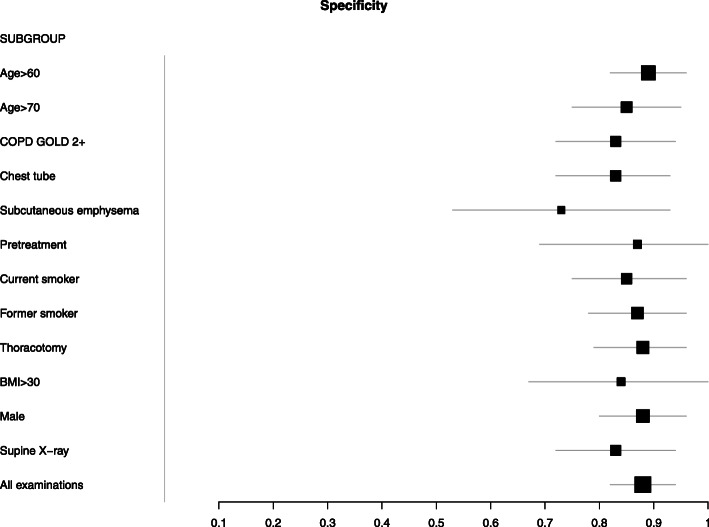
Forest plot of specificity of lung ultrasound in dependence of different covariates. Note that the central dot represents the observed specificity; the true specificity lies between the observed specificity and 1.0 since the reference test (X-ray) is imperfect itself

The sensitivities and specificities for each subgroup are given in Table 1 and illustrated in Figs. 1 and 2. They did not differ between the subgroups (Chi-squared-test for all subgroups, *p* = 0.78 for sensitivity and *p* = 0.62, for specificity).

**Table 1:** Subgroups based on covariates

SUBGROUP	TP	FN	FP	TN	se	95%-CI		*p*	sp	95%-CI		*p*	*N*
Age > 60	30	45	17	140	0,40	0,29	0,51	0.14	0,89	0,82	0,96	0.42	240
Age > 70	17	28	12	66	0,38	0,24	0,51	0.25	0,85	0,75	0,95	0.41	130
COPD GOLD 2+	17	24	15	71	0,41	0,27	0,56	0.66	0,83	0,72	0,94	0.10	140
Chest tube	26	19	15	71	0,58	0,44	0,72	0.04*	0,83	0,72	0,93	0.10	140
Subcutaneous emphysema	6	7	8	22	0,46	0,24	0,69	1	0,73	0,53	0,93	0.02*	52
Pretreatment	8	6	7	47	0,57	0,31	0,83	0.51	0,87	0,69	1	1	68
Current smoker	20	23	10	58	0,47	0,32	0,61	1	0,85	0,75	0,96	0.61	110
Former smoker	21	26	14	95	0,45	0,31	0,58	1	0,87	0,78	0,96	0.96	170
Thoracotomy	28	25	14	100	0,53	0,4	0,66	0.18	0,88	0,79	0,96	1	170
BMI > 30	10	9	10	51	0,53	0,31	0,75	0.66	0,84	0,67	1	0.35	83
Male	29	32	14	106	0,48	0,35	0,6	0.75	0,88	0,8	0,96	0.93	190
Supine X-ray	22	19	15	72	0,54	0,39	0,68	0.25	0,83	0,72	0,94	0.11	140
All examinations	49	59	27	193	0,45	0,36	0,55		0,88	0,82	0,94		340

*TP* true positive, *FN* false negative, *FP* false positive, *TN* true negative, *95%-CI* 95% confidence interval, *se* sensitivity, *sp.* specificity

However, in univariate analysis, subcutaneous emphysema was associated with impaired specificity (73% vs. 88%, *p* = 0.02), and an indwelling chest tube with higher sensitivity (58% vs. 45%, *p* = 0.04). To examine whether the chest tube is confounding with other covariates, we performed the same analyses for both, the sample with an indwelling chest tube (i.e. first day after surgery, *n* = 140) and without (*n* = 200). These data are given as supplementary tables and figures. The above covariates did not influence sensitivity and specificity within the cohorts (chest tube cohort: Chi-squared test for the equality of sensitivities *p* = 0.58, of specificities *p* = 0.58; cohort without chest tube: Chi-squared test for the equality of sensitivities *p* = 1.00, of specificities *p* = 0.86).

### Analysing the false negatives

What are the reasons that ultrasound did not detect a pneumothorax which in turn was present on chest X-ray? There were *n* = 59 false-negative ultrasound examinations; the results of the comparison of patient and surgery related factors of the cohort with false negative and of the remaining cohort are presented in Table 2.

**Table 2:** Comparison of covariates between the subgroup with false-negative ultrasound examination and the remaining population

Covariate	False negatives (*n* = 59)	Rest population (*n* = 274)	*p*-value
	Proportion / mean ± standard deviation	Proportion / mean ± standard deviation	
Age, years	65.7 ± 12.7	64.1 ± 11.0	0.38 ^t^
COPD GOLD grade	mode: 2	mode: 0	0.22 ^c^
Chest tube	0.32	0.42	1 ^c^
Soft tissue emphysema	0.12	0.14	1 ^c^
Ipsilateral pretreatment (surgery or irradiation)	0.10	0.23	1 ^c^
Packyears	33.8 ± 27.35	32.1 ± 26.9	0.66 ^t^
Thoracotomy	0.42	0.52	1 ^c^
BMI, kg/m^2^	25.6 ± 4.6	26.9 ± 5.5	0.08 ^t^
Male gender	0.54	0.55	1 ^c^
Supine X-ray	0.68	0.57	1 ^c^

*c* Chi-squared-test, *t* student‘s t-test

There were no significant differences between the covariates in the false-negative and the remaining sample.

## Discussion

The role of lung ultrasound as an alternative to routine chest roentgenograms for the detection of a pneumothorax after thoracic surgery is under discussion. Two large studies, which were performed under routine [16] or near-routine conditions [23], exhibited a low sensitivity (0.32, 0.19, respectively), whereas two smaller studies with rather artificial conditions (restrictive inclusion criteria [24], small sample size with very high pneumothorax rate [21]) revealed a 1.0 sensitivity. The reasons for this discrepancy are not clear; different sonographic examination techniques and inclusion criteria are suspected to be the cause.

Furthermore, the reported accuracy of postsurgical lung ultrasound is not consistent with the results of large meta-analyses from internal medicine and traumatology, where ultrasound was found to be superior to chest roentgenograms in the detection of a pneumothorax [13–15]. Potential reasons are 1) postoperative anatomic changes like mediastinal shift to the operated side, shrinkage of the operated hemithorax and pleural adhesions which do not regularly occur in medical or trauma patients and 2) that most X-rays in traumatology and intensive care medicine are shot in supine position, impairing the sensitivity of X-ray.

Our results demonstrate that “the usual suspects”, which are considered to debilitate thoracic ultrasound, do not influence the sensitivity of ultrasound under routine conditions. There are various hypotheses why an indwelling chest tube is associated with a higher sensitivity: First, the prevalence of a pneumothorax is higher on the first postoperative day than later. Second, a pneumothorax on the first day after surgery will be larger than later, otherwise one would not have decided to remove the chest tube. We have demonstrated that the sensitivity of ultrasound is dependent on the pneumothorax size [16]. Third, the examiner who – consciously or not – perceives an air leakage through a chest tube will probably expect a pneumothorax and examine more accurately (examiner bias / diagnostic suspicion bias [30, 31]). One should not assume a causal relationship, but also not consider a chest tube as an obstacle to lung ultrasound.

One reason for the wrong assumptions on the prerequisites of lung ultrasound seems to be an insufficient methodology. Most studies, also in internal medicine, assess only or mainly lung sliding to detect or rule out a pneumothorax. While lung sliding is completely specific against a pneumothorax, the inverse conclusion is not true. Absence of lung sliding is found in pleurodesis, fibrosis, COPD, shallow breathing patients (for example after thoracic surgery due to pain), pulmonary emphysema and other pathologic conditions [32]. Relying on lung sliding only leads to a higher false positive rate.

Lung pulse is a powerful, but often neglected artefact to rule out pneumothorax in difficult situations [16, 33].

The addition of B-lines and consolidations increases the accuracy in ruling out a pneumothorax, however, they are not exhaustive in some cases, and there are severe discrepancies in the terminology of “B-lines” and “comet-tail” artefacts [34, 35]. One should be aware of comet tails which mimic B-lines; for example, E-lines are generated by a subcutaneous emphysema and could let an uncareful examiner unjustly rule out a pneumothorax. An insufficient discrimination of different types of comet tails leads to a higher false negative rate. This is consistent with our data, with subcutaneous emphysema being the only covariate which significantly impaired the specificity of lung ultrasound, although we were absolutely aware of the pitfalls of subcutaneous emphysema. The physician who uses ultrasound should indicate radiologic imaging generously if the examination is contradictory or impaired for example by severe skin emphysema.

The reliable detection of a pneumothorax by the lung point can be difficult after thoracic surgery due to altered anatomy or impaired access to the entire hemithorax. In a large pneumothorax, the lung point is not present. Thus, ruling out a pneumothorax by sonography is easier than its detection.

If ultrasound rules out a pneumothorax, no further radiologic imaging is necessary to answer this question, due to the high negative predictive value of ultrasound for a clinically relevant pneumothorax. In contrast, if ultrasound is inconclusive or contradictory to clinical findings, radiologic imaging is obligate. As chest X-ray may also be altered by supine position, obesity, hypoventilation, severe emphysema, etc. in some of these cases a more precise – and more cumbersome – technique is required, which is computed tomography. From the authors’ point of view, ultrasound is a fast and cost-effective tool at the low end of the diagnostic ladder, but also needs training to unfold its full potential.

### Limitations

Taking standard chest roentgenograms as the reference test impairs the calculation of diagnostic accuracy. Staquet et al. [20] described that the sensitivity of a diagnostic index test (ultrasound) can be calculated exactly, if the reference test (roentgenogram) is a hundred per cent specific, which is the case, indeed [13–15]. However, we can only calculate the lower bound of the specificity range. Former trials which were controlled against computed tomography showed that ultrasound is nearly 100% specific for a pneumothorax [19]. Thus, most of “false positives” in the index test are real pneumothoraxes which were overseen by X-ray, i.e. false positives of the reference test. Therefore, we assume that the sensitivity of chest ultrasound should be higher if tested against a perfect reference test (computed tomography).

However, this drawback is the same for every subgroup. Therefore, our data help to find the correct parameters and appropriate patients for ultrasound examination [36]. If forthcoming trials are performed under wrong assumptions, lung ultrasound in thoracic surgery will remain an academic tool for a niche. Furthermore, for certain clinical applications, sensitivity is not the most important test parameter. Two studies have reported the usefulness of lung ultrasound despite its low sensitivity [22, 37]. For instance, the negative predictive value for relevant pneumothorax sizes is clinically more important after chest tube removal, since small apical pneumothoraces are usually not of clinical interest [16, 22].

Particularly after chest tube removal, routine roentgenograms rarely have any consequences (0.9% in 1097 patients, [1]). Bjerregaard et al. therefore recommend an individualised approach based on clinical information. Ultrasound could be such a tool to significantly reduce the number of postoperative roentgenograms.

## Conclusion

The conditions usually considered inappropriate for lung ultrasound should not be excluded in forthcoming diagnostic accuracy studies on postoperative lung ultrasound. They can be mastered with a thorough sonographic technique. However, in the case of a severe subcutaneous emphysema, radiologic imaging should be indicated generously. For the majority of patients, ultrasound could be the first step of an escalating approach where X-ray is only performed on demand; this needs to be further evaluated under “real world conditions”. Instead of constructing artificial study environments, the investigators’ efforts should concentrate on improving the sonographic technique to meet those “difficult” conditions and the pathoanatomic and pathophysiologic particularities of patients shortly after thoracic surgery.

## Supplementary Information

**Additional file 1: Supplementary figure** Forest plots of sensitivity (left) and specificity (right side) of lung ultrasound for pneumothorax in the cohort without (top) and with indwelling chest tubes (bottom). Note that the observed specificity is illustrated; the true specificity lies between the observed specificity and 1.0 since the reference test (X-ray) is imperfect itself.

**Additional file 2: Supp Table 1.** Subgroups based on covariates for the cohort without chest tubes. TP: true positive. FN: false negative. FP: false positive. TN: true negative. 95%-CI: 95% confidence interval. se: sensitivity. sp: specificity.

**Additional file 3: Supp Table 2**. Subgroups based on covariates for the cohort with chest tubes (on the first postoperative day). TP: true positive. FN: false negative. FP: false positive. TN: true negative. 95%-CI: 95% confidence interval. se: sensitivity. sp: specificity

## Data Availability

The datasets generated and/or analysed during the current study are not publicly available due data privacy concerns, but an anonymised minimal data set is available from the corresponding author on reasonable request. All data which are necessary to control the results are given within the manuscript.

## Abbreviations

a.p./p.a.: Anterior-posterior/posterior-anterior
BMI: Body mass index
COPD: Chronic obstructive pulmonary disease
ECG: Electrocardiogram
EDC: Electronic data capture
FN/FP: False negative/ false positive
GOLD: The Global Initiative for Chronic Obstructive Lung Disease
se: Sensitivity
sp.: Specificity
TN/TP: True negative/ true positive
WHO: World Health Organisation
