# Supplementary figures and images for: Most patient conditions do not a priori debilitate the sensitivity of thoracic ultrasound in thoracic surgery-a prospective comparative study
Source: J Cardiothorac Surg. 2021 Apr 13;16:75. doi: 10.1186/s13019-021-01454-6 (PMC8045207; doi:10.1186/s13019-021-01454-6)

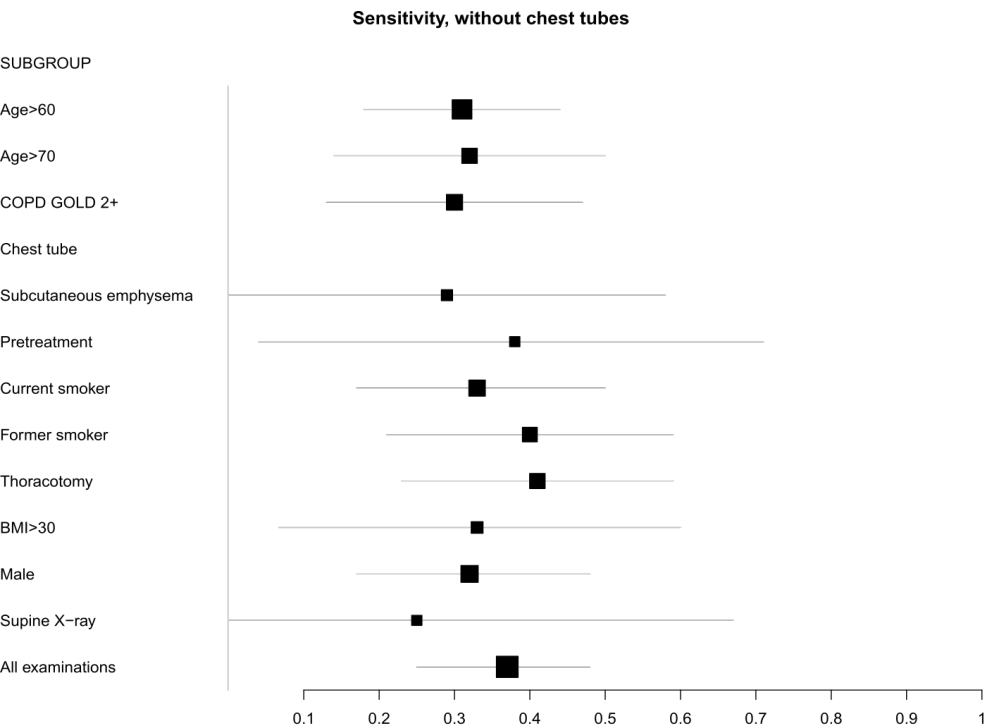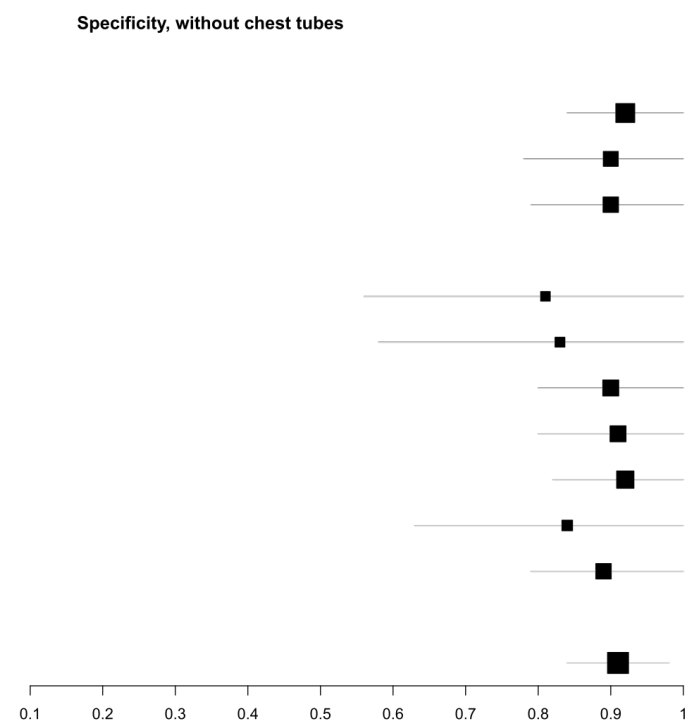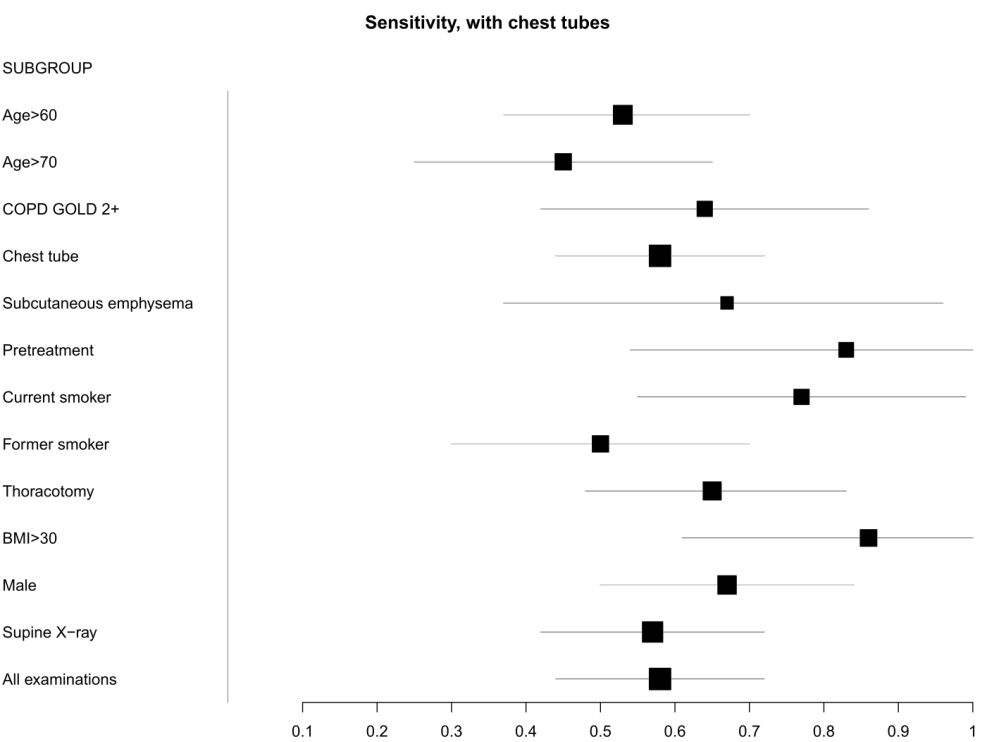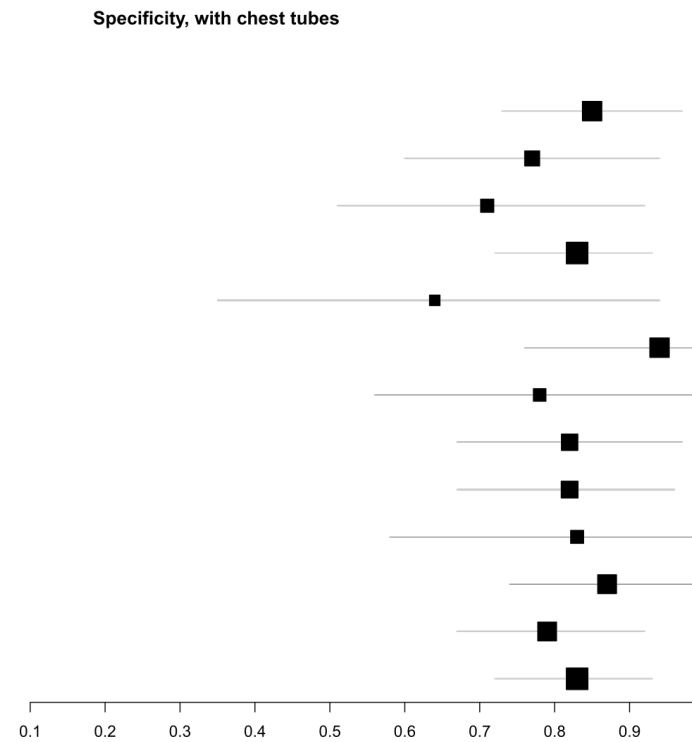

Supplement: Supplementary file 1 — Additional file 1: Supplementary figure Forest plots of sensitivity (left) and specificity (right side) of lung ultrasound for pneumothorax in the cohort without (top) and with indwelling chest tubes (bottom). Note that the observed specificity is illustrated; the true specificity lies between the observed specificity and 1.0 since the reference test (X-ray) is imperfect itself. [file 13019_2021_1454_MOESM1_ESM.pdf]
