# Supplementary material for: Most patient conditions do not a priori debilitate the sensitivity of thoracic ultrasound in thoracic surgery-a prospective comparative study
Source: J Cardiothorac Surg. 2021 Apr 13;16:75. doi: 10.1186/s13019-021-01454-6 (PMC8045207; doi:10.1186/s13019-021-01454-6)
Supplement: Supplementary file 2 — Additional file 2: Supp Table 1. Subgroups based on covariates for the cohort without chest tubes. TP: true positive. FN: false negative. FP: false positive. TN: true negative. 95%-CI: 95% confidence interval. se: sensitivity. sp: specificity. [file 13019_2021_1454_MOESM2_ESM.docx]

| SUBGROUP | TP | FN | FP | TN | se | 95%-CI | | p | sp | 95%-CI | | p | N |
| --- | --- | --- | --- | --- | --- | --- | --- | --- | --- | --- | --- | --- | --- |
| Age>60 | 14 | 31 | 8 | 88 | 0,31 | 0,18 | 0,44 | 0.26 | 0,92 | 0,84 | 1 | 0.95 | 140 |
| Age>70 | 8 | 17 | 5 | 43 | 0,32 | 0,14 | 0,5 | 0.74 | 0,9 | 0,78 | 1 | 0.90 | 74 |
| COPD GOLD 2+ | 8 | 19 | 5 | 46 | 0,3 | 0,13 | 0,47 | 0.47 | 0,9 | 0,79 | 1 | 1 | 80 |
| Subcutaneous emphysema | 2 | 5 | 3 | 13 | 0,29 | 0 | 0,58 | 0.96 | 0,81 | 0,56 | 1 | 0.32 | 27 |
| Pretreatment | 3 | 5 | 6 | 30 | 0,38 | 0,04 | 0,71 | 1 | 0,83 | 0,58 | 1 | 0.12 | 44 |
| Current smoker | 10 | 20 | 4 | 37 | 0,33 | 0,17 | 0,5 | 0.81 | 0,9 | 0,8 | 1 | 1 | 73 |
| Former smoker | 10 | 15 | 6 | 59 | 0,4 | 0,21 | 0,59 | 0.84 | 0,91 | 0,8 | 1 | 1 | 93 |
| Thoracotomy | 11 | 16 | 5 | 60 | 0,41 | 0,23 | 0,59 | 0.73 | 0,92 | 0,82 | 1 | 0.85 | 94 |
| BMI>30 | 4 | 8 | 6 | 31 | 0,33 | 0,067 | 0,6 | 1 | 0,84 | 0,63 | 1 | 0.14 | 50 |
| Male | 11 | 23 | 8 | 67 | 0,32 | 0,17 | 0,48 | 0.63 | 0,89 | 0,79 | 1 | 0.63 | 110 |
| Supine X-ray | 1 | 3 | 0 | 14 | 0,25 | 0 | 0,67 | 1 | 1 | 1 | 1 | 0.46 | 18 |
| All examinations | 23 | 40 | 12 | 122 | 0,37 | 0,25 | 0,48 |  | 0,91 | 0,84 | 0,98 |  | 200 |
