# Supplementary material for: Most patient conditions do not a priori debilitate the sensitivity of thoracic ultrasound in thoracic surgery-a prospective comparative study
Source: J Cardiothorac Surg. 2021 Apr 13;16:75. doi: 10.1186/s13019-021-01454-6 (PMC8045207; doi:10.1186/s13019-021-01454-6)
Supplement: Supplementary file 3 — Additional file 3: Supp Table 2. Subgroups based on covariates for the cohort with chest tubes (on the first postoperative day). TP: true positive. FN: false negative. FP: false positive. TN: true negative. 95%-CI: 95% confidence interval. se: sensitivity. sp: specificity [file 13019_2021_1454_MOESM3_ESM.docx]

| SUBGROUP | TP | FN | FP | TN | se | 95%-CI | | p | sp | 95%-CI | | p | N |
| --- | --- | --- | --- | --- | --- | --- | --- | --- | --- | --- | --- | --- | --- |
| Age>60 | 16 | 14 | 9 | 52 | 0,53 | 0,37 | 0,7 | 0.59 | 0,85 | 0,73 | 0,97 | 0.48 | 99 |
| Age>70 | 9 | 11 | 7 | 23 | 0,45 | 0,25 | 0,65 | 0.21 | 0,77 | 0,6 | 0,94 | 0.45 | 57 |
| COPD GOLD 2+ | 9 | 5 | 10 | 25 | 0,64 | 0,42 | 0,86 | 0.79 | 0,71 | 0,51 | 0,92 | 0.05 | 56 |
| Subcutaneous emphysema | 4 | 2 | 5 | 9 | 0,67 | 0,37 | 0,96 | 0.98 | 0,64 | 0,35 | 0,94 | 0.11 | 25 |
| Pretreatment | 5 | 1 | 1 | 17 | 0,83 | 0,54 | 1 | 0.36 | 0,94 | 0,76 | 1 | 0.25 | 24 |
| Current smoker | 10 | 3 | 6 | 21 | 0,77 | 0,55 | 0,99 | 0.19 | 0,78 | 0,56 | 1 | 0.63 | 41 |
| Former smoker | 11 | 11 | 8 | 36 | 0,5 | 0,3 | 0,7 | 0.46 | 0,82 | 0,67 | 0,97 | 1 | 73 |
| Thoracotomy | 17 | 9 | 9 | 40 | 0,65 | 0,48 | 0,83 | 0.37 | 0,82 | 0,67 | 0,96 | 1 | 80 |
| BMI>30 | 6 | 1 | 4 | 20 | 0,86 | 0,61 | 1 | 0.23 | 0,83 | 0,58 | 1 | 1 | 33 |
| Male | 18 | 9 | 6 | 39 | 0,67 | 0,5 | 0,84 | 0.24 | 0,87 | 0,74 | 0,99 | 0.44 | 77 |
| Supine X-ray | 21 | 16 | 15 | 58 | 0,57 | 0,42 | 0,72 | 1 | 0,79 | 0,67 | 0,92 | 0.16 | 120 |
| All examinations | 26 | 19 | 15 | 71 | 0,58 | 0,44 | 0,72 |  | 0,83 | 0,72 | 0,93 |  | 140 |
